# Tributyltin Exposure Is Associated With Recognition Memory Impairments, Alterations in Estrogen Receptor α Protein Levels, and Oxidative Stress in the Brain of Female Mice

**DOI:** 10.3389/ftox.2021.654077

**Published:** 2021-04-09

**Authors:** Igor Ferraz da Silva, Eduardo Merlo, Charles S. Costa, Jones B. Graceli, Lívia C. M. Rodrigues

**Affiliations:** ^1^Laboratory of Neurotoxicology and Psychopharmacology, Department of Physiological Sciences, Federal University of Espírito Santo, Vitoria, Brazil; ^2^Laboratory of Endocrinology and Cellular Toxicology, Department of Morphology, Federal University of Espírito Santo, Vitoria, Brazil

**Keywords:** tributyltin, recognition memory, estrogen, oxidative stress, prefrontal cortex, hippocampus

## Abstract

Tributyltin (TBT) is a persistent organometallic pollutant widely used in several agricultural and industrial processes. TBT exposure is associated with various metabolic, reproductive, immune, and cardiovascular abnormalities. However, few studies have evaluated the effects of TBT on behavior. In the present study, we aimed to investigate whether TBT exposure results in oxidative, neuroendocrine, and behavioral alterations. TBT was administered to adult female mice (250, 500, or 750 ng/kg/day or veh for 14 days), and their recognition memory was assessed. We have also evaluated estrogen receptor (ER)α protein expression and oxidative stress (OS) in brain areas related to memory, as well as the correlation between them. A reduction in short- and long-term recognition memory (STM and LTM) performance, as well as in total exploration time was observed in TBT mice. Reduced ERα protein expression was observed in the prefrontal cortex (PFC) and hippocampus of TBT mice, while an increase in TBARS concentration was observed in the PFC of treated animals. Collectively, these data suggest that TBT exposure impairs recognition memory in female mice as a result of, at least in part, its toxicological effects on ERα expression and OS in specific brain areas related to memory.

## Introduction

Organotins (OTs), such as tributyltin (TBT), are organometallic pollutants that can act as endocrine-disrupting chemicals (EDCs), interfering with normal metabolic, reproductive, and other endocrine functions (Omura et al., 2001; Grote et al., 2004). TBT also exerts other deleterious effects, such as cytotoxic, genotoxic, and neurotoxic actions in several invertebrate and vertebrate experimental models (Ahmad et al., 2008; Mitra et al., 2013; Ferraz da Silva et al., 2018). TBT induces imposex, the abnormal induction of male sex characteristics in female gastropod mollusks, leading to reproductive abnormalities. Since the 1960's, the most notable use for TBT has been as an antifouling agent in paints for marine ships and fishing nets (ten Hallers-Tjabbes et al., 1994). TBT has an extensive environmental half-life and is highly susceptible to bioaccumulation, therefore facilitating human exposure primarily due to contaminated seafood, water ingestion, and sediments (Kannan et al., 1995; Chien et al., 2002).

Several studies have reported that consumption of seafood containing OTs is the primary source of human exposure in different continents around the world (Toyoda et al., 2000; Rantakokko et al., 2006; Merlo et al., 2018). OTs have been detected in human blood samples in the USA at levels that range from 64 to 155 ng/mL and in human liver samples from Poland at levels that range from 2.4 to 11 ng/g, which indicates TBT accumulation in tissues (Whalen et al., 1999; Kannan and Falandysz, 2017). Our previous studies have reported an increase in tin levels in blood and/or organs after TBT exposure (100 ng/kg/day) for 15 days, leading to metabolic, cardiovascular, and reproductive abnormalities in female rats (Bertuloso et al., 2015; Coutinho et al., 2016; Sena et al., 2017). Penza et al. (2011) reported that TBT surprisingly leads to estrogen receptor activation in adipocytes at doses similar to the estimated human intake. Estrogenic effects, however, varied with exposure time, sex, and dose, indicative of the complex nature of TBT toxicity. Mitra et al. (2013) reported that TBT can disrupt the blood-brain barrier, inducing oxidative stress and neuronal cell death, initiating neurodegeneration within 3–7 days after a single exposure in rats.

Previous studies have shown that estrogen (E2) presents key neuroprotective, cognitive, and antioxidant roles, acting mainly through the nuclear estrogen receptors (ER) ERα and ERβ (Nilsen et al., 2006; Zhao and Brinton, 2006; Walf et al., 2011; Mosquera et al., 2014). The role of ERs in proper memory and learning is supported by their distribution in the prefrontal cortex (PFC), hippocampus (HCP), as well as in other brain regions related to cognition (Kolb, 1984; Frick et al., 2010; Shanmugan and Epperson, 2014; Almey et al., 2015). Although ERβ has fundamental roles in mediating neuroprotection through antioxidant activity (Sharma et al., 2017), ERα is the predominant ER in the mouse hippocampus and PFC, playing an important role in their normal function (Mitra et al., 2003; Bailey et al., 2011). However, it is not well-understood if TBT disrupts ERα levels, and subsequently E2 action in those brain regions, possibly impairing memory function as a result.

Since the discovery of the EDC effects of TBT, few studies have reported its toxicological effect on brain regions responsible for learning and memory (Mitra et al., 2013, 2015). In the present study, we hypothesized that TBT leads to recognition memory abnormalities, which could be a result from impaired ERα expression and oxidative stress (OS). We analyzed key indicators of behavioral and brain function, including short- and long-term recognition memory (STM and LTM), ERα expression, and OS in the PFC and hippocampus, and the possible correlation between these indicators. Identifying altered recognition memory performance associated with TBT exposure and the potential underlying mechanisms may contribute to our continuously evolving understanding of the brain targets for EDCs.

## Materials and Methods

### Subjects and Treatment

Sixty-four female Swiss mice aged 8 weeks old in the beginning of the experiments were used in this study, having been obtained from the institutional animal care facility of the Federal University of Espírito Santo. The chosen age is the age when female mice present regular estrous cycles. The animals were housed in groups of five per cage in a room with controlled temperature (22 ± 1°C) and 12:12 h light/dark cycle with free access to commercial standard chow and water. All animals maintained good health indicators and did not present variation in weight during the experiments and after the protocols were finished. Three eventual deaths occurred during experiments for reasons unrelated to the treatment. This study was approved by the local Ethics Committee of Animal Use for Research under the protocol number 33/2013, in accordance with the guidelines of the National Council for Animal Experiments Control (CONCEA). Tributyltin chloride (TBT, 96%; Sigma) was dissolved in ethanolic solution (0.1%).

### Procedures and Study Design

Mice from different litters were randomly divided into the following four groups: (1) control (CON) mice were treated daily with vehicle (0.1% ethanol solution); (2) TBT250 (250 ng/kg/day); (3) TBT500 (500 ng/kg/day), and (4) TBT750 (750 ng/kg/day). For all treatment groups, mice were treated intragastrically (i.g.) at a dose of 1 ml/100 g for 14 days. At the end of the experiments, the animals were anesthetized with ketamine (20 mg/kg) and xylazine (10 mg/kg) solutions (0.2 and 0.1 ml/kg, i.p., respectively), and euthanized by decapitation at the same phase of the estrous cycle (metaestrus-diestrus), noting that some animals in the control group were euthanized a day after to ensure that they have reached metaestrus-diestrus. Prefrontal cortex (PFC) and hippocampus (HCP) samples were collected from the same animals that went through behavioral testing and stored at −80°C until analysis.

The lowest TBT dose employed here (250 ng/kg) is below 300 ng/kg, the value considered safe for humans by the United States Environmental Protection Agency (1997) and the World Health Organization (WHO (World Health Organization), 1990). The intermediary TBT dose used (500 ng/kg) is close to the estimated human intake (0.5 μg/kg) (Penza et al., 2011). The highest TBT dose was chosen considering the TBT levels found in sea sediment and mollusk tissue along the Brazilian coast, even after restrictions in the legal use of TBT (Almeida et al., 2004; Sant'Anna et al., 2014; Artifon et al., 2016; Maciel et al., 2018). In addition, TBT doses and oral route of exposure were selected taking into consideration previous studies that demonstrated toxicity on the metabolic, cardiovascular, and other systems (Penza et al., 2011; Coutinho et al., 2016; Ribeiro-Júnior et al., 2016).

### Estrous Cycle Evaluation

TBT leads to abnormal estrous cyclicity (Podratz et al., 2012). Thus, we determined the stage of the estrous cycle during the 14 days of TBT exposure as reported previously (Nelson et al., 1982). Briefly, vaginal smears were collected daily between 9:30 and 10:00 A.M. The smears were stained with hematoxylin and eosin (H&E) and assessed under a microscope. The estrous cycle stage was classified as proestrus (P), estrus (E), or metaestrus-diestrus (M-D) based on the observed ratios of cornified epithelial cells, nucleated epithelial cells, and polymorphonuclear leukocytes. The frequencies of the estrous cycles and the days spent in the different phases were compared among the experimental groups (*n* = 4–6; Supplementary Figures 1A,B).

### Novel Object Recognition Task

To evaluate recognition memory and object exploration behavior, we performed an adaptation of object recognition test as proposed by Ennaceur and Delacour (1988). In the morning of the last day of treatment, we performed the novel object recognition test. The animals were habituated for 10 min in an acrylic square box of 350 × 350 × 300 mm (length × width × height) dimensions, with the floor covered with shavings and the walls blinded with white opaque paper. After 24 h, animals were re-exposed to the box, and two equal objects [first objects (OOs)] were introduced into diagonal corners and left for 5 min (pre-test). Short-term memory (STM) was assessed 90 min after the pre-test. In this test session, one of the OOs was replaced with a novel object (NO), and the animals were exposed to the two objects for 5 min. Long-term memory (LTM) was assessed 24 h after the pre-test; the NO was replaced with a substitute object (SO), and the animals were exposed to the objects for 5 min. Test sessions were recorded and analyzed with the ANY-maze behavioral tracking software version 4.99 (Stoelting Co., Wood Dale, USA), and the recognition index obtained according to the following formulas: [time NO × 100/(time NO + time OO)] for short-term memory, and [time SO × 100/(time SO + time OO)] for long-term memory. After data analysis, a recognition index below 60% was defined as cognitive deficit as previously reported (Ennaceur and Delacour, 1988). For exploration behavior analysis, total exploration time was calculated as (time NO + time SO + time OO).

### Protein Extraction and Immunoblotting

PFC and hippocampus were collected and total protein levels obtained following the protocol presented in Bertuloso et al. (2015). Briefly, PFC and hippocampus protein samples were loaded onto an SDS/PAGE gel for immunoblotting analysis (Bio-Rad). Primary antibodies included anti-ERα (ERα, sc7207; 1:500, Santa Cruz Biotechnology, Santa Cruz, CA) and glyceraldehyde 3-phosphate dehydrogenase (GAPDH) (sc25778, 1:1250, Santa Cruz Biotechnology). ERα and GAPDH proteins were detected using a secondary anti-rabbit IgG alkaline phosphatase conjugate (sc-2007, 1:1000, Santa Cruz Biotechnology). Blots for ERα and their respective GAPDH control were visualized using a color development reaction containing BCIP/NBT solution (sc24981, Santa Cruz Biotechnology). Protein bands were analyzed by densitometry using ImageJ software. Relative expression levels were normalized by dividing the values of the protein of interest by the corresponding internal control values. Total protein level was performed at the LABIOM Laboratory, UFES, Brazil.

### Oxidative Stress Assessment

Oxidative stress was assessed using the thiobarbituric acid reactive substance (TBARS) assay as previously described (Coutinho et al., 2016). Briefly, PFC and hippocampus samples were mixed with 1 ml of 10% trichloroacetic acid and 1 ml of 0.67% thiobarbituric acid; subsequently, the samples were heated in a boiling water bath for 15 min. TBARS levels were determined by absorbance at 530 and 600 nm and expressed as nanomoles per gram, calculated from a standard curve using standard dilutions.

### Statistical Analysis

All data are presented as mean ± SEM. Sample size and power calculations were performed using G^*^Power 3.1.9.6 (Universität zu Kiel, Germany). For each data set, a D'Agostino-Pearson omnibus normality test was also performed. If the aforesaid test revealed non-Gaussian data, a Kruskal-Wallis followed by Dunn's multiple comparisons test was then used. If the data passed the normality test, the one-way ANOVA followed by Bonferroni's *post-hoc* test for multiple comparisons was applied. To evaluate the relationship between the assessed parameters (Supplementary Figure 2), Spearman's or Pearson's correlation was used for non-Gaussian or Gaussian distributions, respectively. All correlations were obtained from paired animal values. Finally, when statistical significance was identified, we tested whether linear or non-linear regression was a better fitting. A value of *p* < 0.05 was considered statistically significant. Statistical analyses and graphical constructions were performed using GraphPad Prism version 7.00 (La Jolla, CA, USA).

## Results

### TBT Mice Have Abnormal Estrous Cycles

Vaginal smears were collected daily for 14 days and examined under an optical microscope for the evaluation of the estrous cycle stage (*n* = 4–6; Supplementary Figures 1A,B). TBT250, TBT500, and TBT750 mice displayed abnormal and longer estrous cycles and spent more days in the M-D phase (~60% of the time) compared with CON mice [*p* < 0.01, *F*_(3, 12)_ = 11.17; Supplementary Figures 1A,B]. No significant differences were observed as to duration of each estrous cycle phase and total cycle length between the different TBT-treated groups (*p* > 0.05; Supplementary Figures 1A,B).

### TBT Mice Present Impaired Short- and Long-Term Recognition Memory

**Figure 1:**
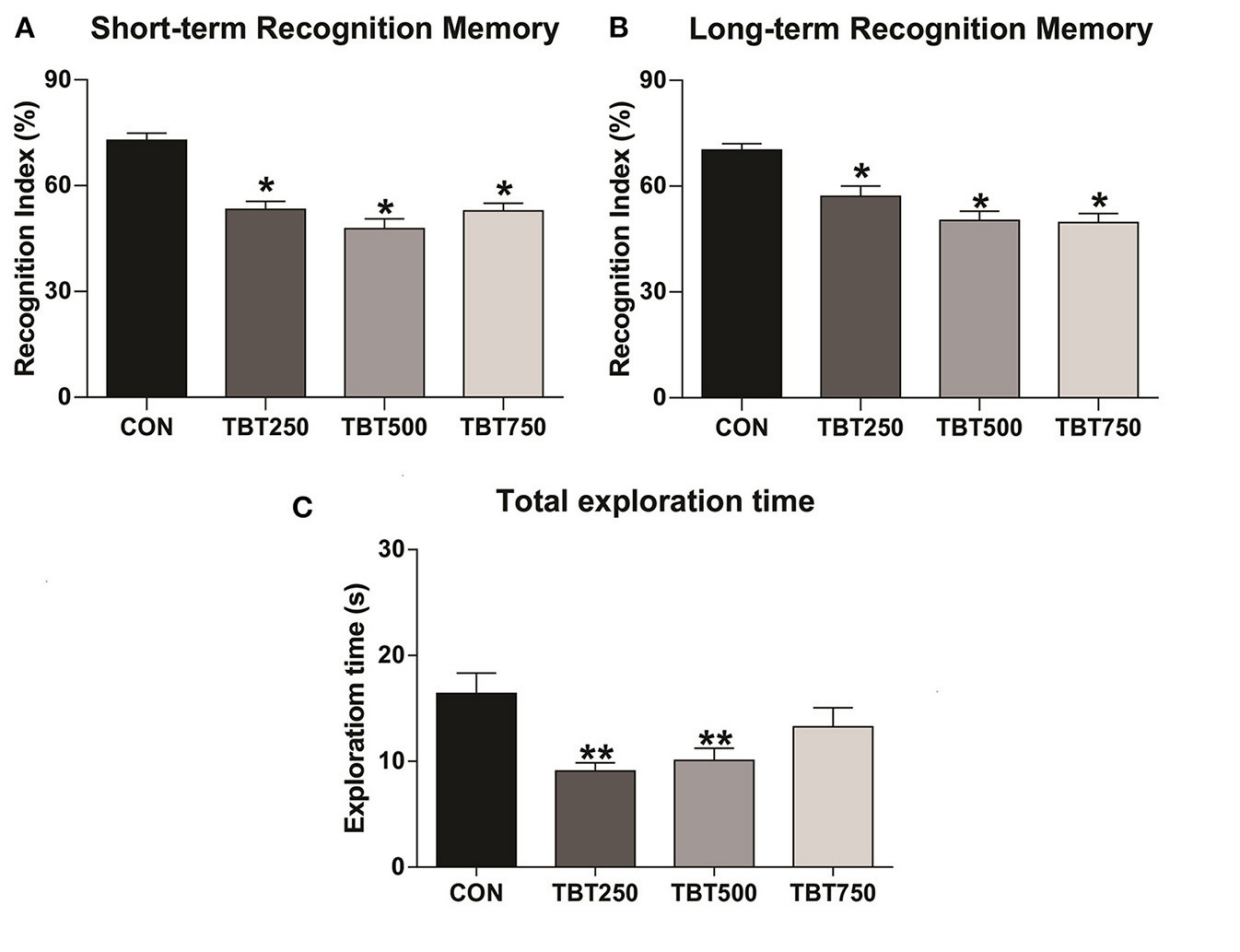
TBT exposure leads to abnormal recognition memory in female mice. Short- **(A)** and long-term **(B)** recognition memory performance (STM and LTM, respectively). **(C)** TBT exposure results in abnormal total exploration time in female mice. A reduction in total exploration time was observed in TBT-treated mice. *p < 0.05 vs. CON; **p < 0.01 vs. CON (one-way ANOVA, followed by Bonferroni's test).

TBT was capable of altering cognitive parameters in female mice (Figure 1). Following the paradigm of Ennaceur and Delacour (1988), STM and LTM were assessed by substituting one of the previous objects with a novel object, and mice were exposed to two new objects in different time frames (1.5 and 24 h for STM and LTM, respectively). TBT impaired recognition memory on both time parameters in all doses tested here, as the three treated groups exhibited reduced recognition indexes when compared to the control group. Regarding STM, when compared with the control [*p* < 0.05, *F*_(3, 12)_ = 10.30], the TBT-exposed mice failed to achieve a mean recognition index equal or above 60%, indicative of cognitive deficit in all tested doses (CON: 70 ± 8%, TBT250: 59.25 ± 6%, TBT500: 44.25 ± 10%, TBT750: 47.75 ± 8%, *n* = 12; Figure 1A). No significant differences in STM were observed among the TBT-treated groups (*p* > 0.05), as shown in Figure 1A. As to LTM, a reduction in the recognition index was observed in all TBT groups compared with the control mice [*p* < 0.05 *F*_(3, 12)_ = 7.947], and again, TBT treatment prevented the groups from achieving a mean recognition index of 60% in all doses analyzed here (CON: 72.5 ± 6%, TBT250: 56.25 ± 7%, TBT500: 43.75 ± 8%, TBT750: 45.75 ± 9%, *n* = 12; Figure 1B). No significant differences in LTM were observed among the TBT-treated groups (*p* > 0.05; Figure 1B).

### TBT Mice Present a Reduction in Object Exploration

In addition to evaluating the recognition memory of the subjects, we have also analyzed the effects of TBT on spontaneous exploration, as this behavior is linked to overall cognitive performance (Gangadharan et al., 2016). A reduced [*p* < 0.05, *F*_(3, 21)_ = 4.431] total object exploration time, in seconds, was observed in the TBT250 and TBT500 groups compared with the control mice (CON: 16.41 ± 1.91 s, TBT250: 9.08 ± 0.78 s, TBT500: 10.08 ± 1.51 s, TBT750: 13.27 ± 1.78 s, *n* = 12; Figure 1C). No significant statistical difference was observed between CON and TBT750 mice (*p* > 0.05; Figure 1C). Moreover, no significant differences in total exploration time were observed between TBT-treated groups (*p* > 0.05; Figure 1C).

### TBT Decreased ERα Protein Expression in Brain Regions Related to Recognition Memory

**Figure 2:**
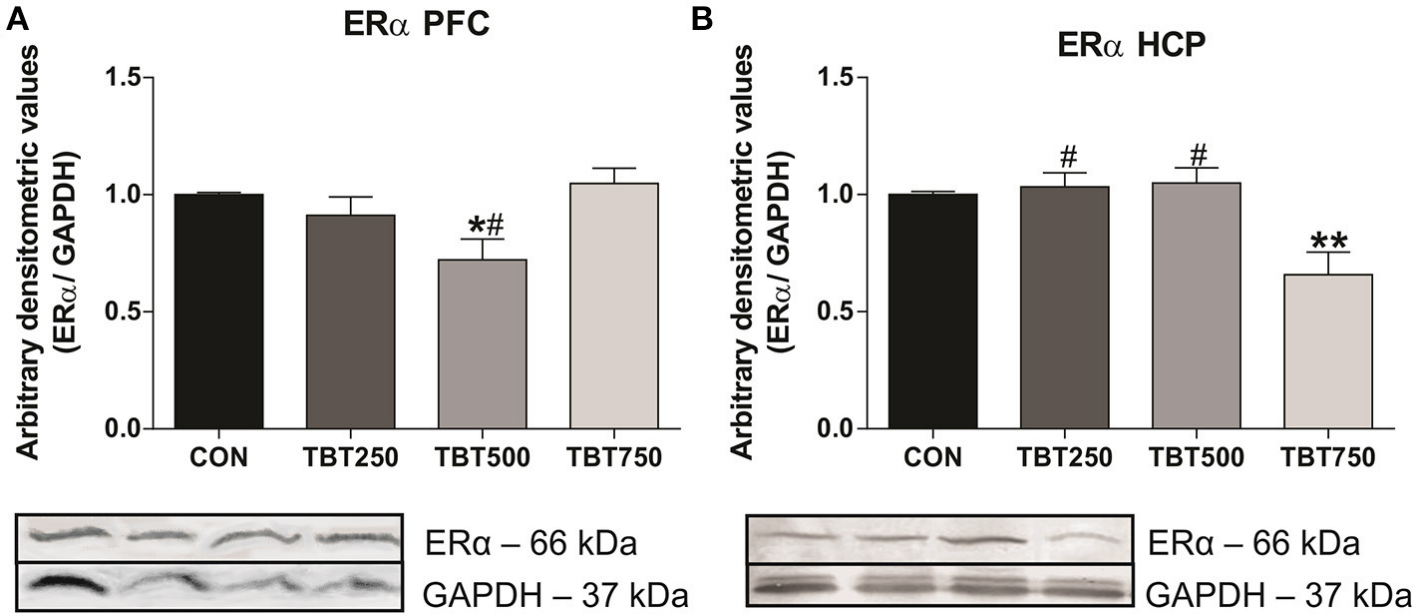
TBT exposure induced abnormal ERα protein expression in brain areas of female mice. Reduced ERα protein expression in the PFC **(A)** and hippocampus (HCP) **(B)** from female mice. *p < 0.05 vs. CON; **p < 0.01 vs. CON; #*p* < 0.05 vs. TBT750 (one-way ANOVA, followed by Bonferroni's test).

ERα protein expression in the PFC and hippocampus was evaluated using immunoblotting analysis. Although no significant differences in ERα protein expression were observed between CON, TBT250, and TBT750 mice in the PFC (*n* = 4–6, *p* > 0.05; Figure 2A), it was found to be reduced by 28% in TBT500 [*F*_(3, 15)_ = 4.840] compared with control mice (*n* = 4–6, *p* < 0.05; Figure 2A). No significant differences in ERα protein expression were observed between TBT250 and TBT750 mice in the PFC (*n* = 4–6, *p* > 0.05; Figure 2A), but it was found to be reduced in TBT500 mice when compared with the TBT750 group (*n* = 4–6, *p* < 0.05; Figure 2A).

In addition, no significant differences in ERα protein expression were observed between CON, TBT250, and TBT500 mice in the hippocampus (*n* = 4–6, *p* > 0.05; Figure 2B). A 35% reduction in ERα protein expression was observed in the hippocampus from TBT750 mice [*F*_(3, 11)_ = 8.100] compared with control mice (*n* = 4–6, *p* < 0.05; Figure 2B). No significant difference was observed between TBT250 and TBT500 mice (*n* = 4–6, *p* > 0.05; Figure 2B). In addition, ERα protein expression in TBT750 mice was lower when compared with TBT250 and TBT500 mice in this brain region (*n* = 4–6, *p* < 0.05; Figure 2B).

### TBT Mice Have Increased TBARS Levels in a Brain Region Related to Memory

**Figure 3:**
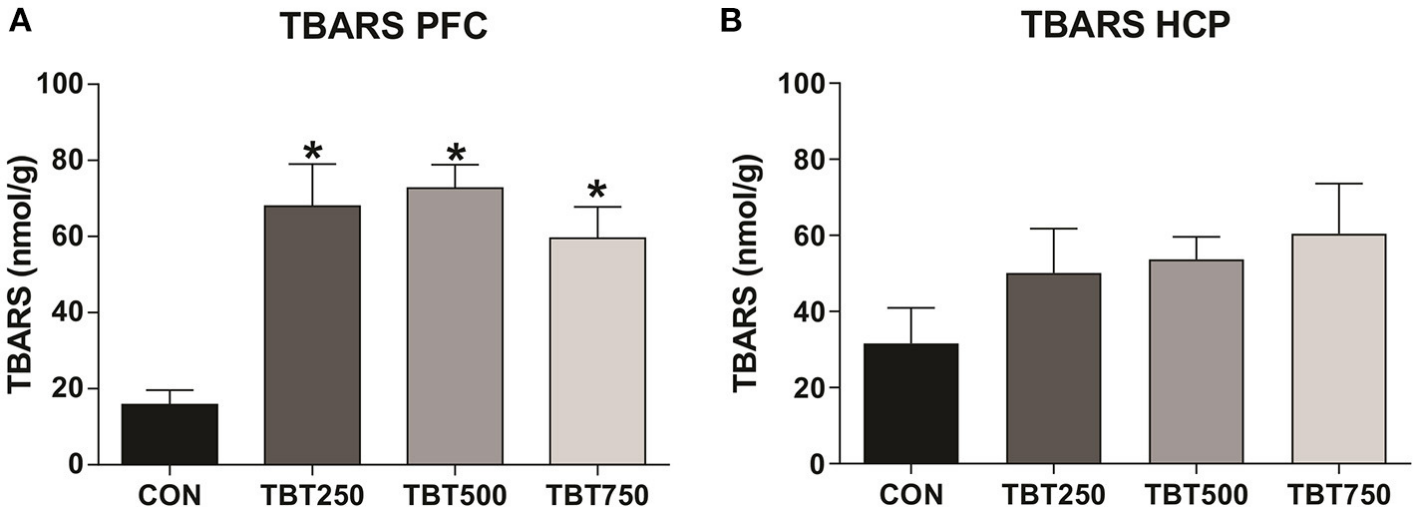
TBT exposure increases oxidative stress in brain areas of female mice. Increased PFC **(A)** and hippocampus (HCP) **(B)** TBARS levels. *p < 0.05 vs. CON (one-way ANOVA, followed by Bonferroni's test).

Oxidative stress in the PFC and hippocampus samples was assessed using the TBARS assay. An increase in TBARS levels was observed in the PFC of all TBT treatment groups when compared with the control mice, an indicator of oxidative stress [CON: 15.80 ± 3.81, TBT250: 65.92 ± 11.08, TBT500: 72.74 ± 6.13, TBT750: 59.57 ± 8.18; *p* < 0.01, *F*_(3, 8)_ = 7.778]. No significant differences in TBARS levels were observed between TBT250, TBT500, and TBT750 mice in the PFC (*n* = 4–6, *p* > 0.05; Figure 3A). In addition, no significant differences in TBARS levels were observed between CON, TBT250, TBT500, and TBT750 groups in the hippocampus [*n* = 4–6, *p* > 0.05, *F*_(3, 10)_ = 1.309; Figure 3B].

## Discussion

**Figure 4:**
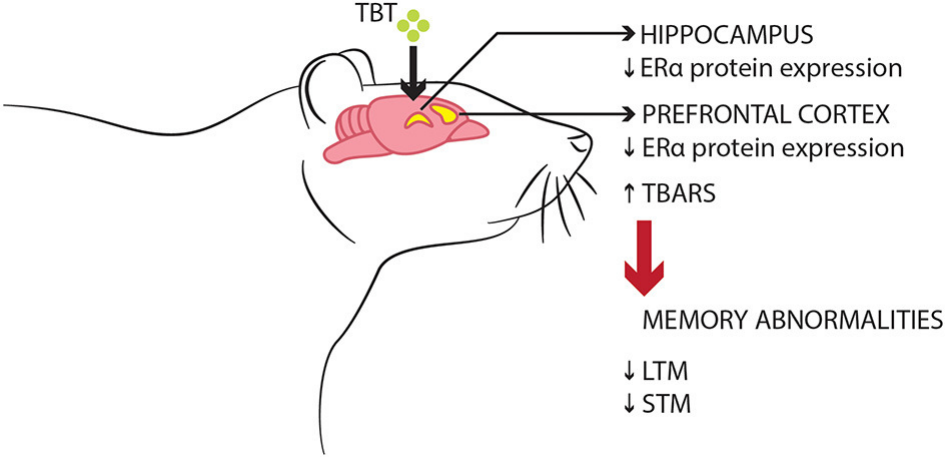
Schematic model of the suggested deleterious effects of TBT on memory function in female mice. TBT exposure for 14 days resulted in recognition memory impairments, disrupted ERα expression, and oxidative stress in brain regions such as the PFC and hippocampus.

Initial observations from this present study suggest that TBT exposure may lead to recognition memory impairments in both short- and long-term parameters, alongside altering object exploration behavior, disrupting ERα protein expression in the PFC and hippocampus, and increasing the levels of TBARS, an oxidative marker, in the PFC of female mice. Therefore, these data suggest that the toxicological effects of TBT in the PFC could represent one of many potential mechanisms underlying recognition memory impairments in female mice (Figure 4).

As reported in our previous studies, TBT exposure leads to reproductive complications due to abnormal steroidogenesis, impaired ER expression in the reproductive tract, disrupted estrous cyclicity, improper function of the hypothalamic pituitary gonadal (HPG) axis, and irregular modulation of other signaling networks (Podratz et al., 2012, 2015; Sena et al., 2017; de Araújo et al., 2018). Podratz et al. (2012) and Sena et al. (2017) reported irregular estrous cyclicity in TBT-treated animals, with a longer metaestrus-diestrus (MD) phase and lower serum E2 levels having been observed in female rats exposed to TBT (100 ng/kg) for 2 weeks. Our current findings of irregular estrous cyclicity, increased cycle length, and longer MD phase in all TBT groups are consistent with those previous reports and further demonstrate the detrimental effects of TBT on reproductive function (Supplementary Figure 1). We opted for using only female mice in this study to better evaluate if the classic endocrine-disrupting effects of TBT on reproduction, such as irregular estrous cycles, were concomitant with alterations in behavior and the brain, but a model with males and a sex-dependent analysis is on our plans for a future study.

A few studies have assessed brain abnormalities induced by TBT in a rodent model (Mitra et al., 2013, 2014, 2015). These important studies reported neuronal apoptosis, oxidative stress, and neurodegenerative processes in the brain of male rats on days 3 and 7 after a single treatment with TBT (10, 20, and 30 mg/kg). However, these studies have not investigated behavioral processes, such as recognition memory and object exploration time as presented here, or the effects of TBT in specific cortical areas involved in memory function, such as the PFC and hippocampus. Our current data demonstrate that STM and LTM performances in Novel Object Recognition Task (NORT) were affected in all doses, suggesting an impairment in recognition memory of mice exposed to TBT for 2 weeks. Total exploration time were affected as well, but no statistically significant reduction was detected in the group TBT750, which suggests a subtle biphasic response, with a slightly J-shaped curve. Such dose-response pattern is commonly found in toxicological studies (Calabrese, 2013), and it can happen due to many biological variables that our current model cannot account for, besides the documented non-linear behavior of EDC toxicity curves (Vandenberg et al., 2012).

Our data also showed a reduction in ERα protein expression in the PFC and hippocampus of TBT-treated mice (Figure 2). The results indicate that TBT does not induce a linear dose-response relationship, with higher doses not being able to lead to a significant reduction in protein expression in PFC, while the contrary was detected in hippocampus. Although our current model cannot specifically predict on which exact pathways TBT acts to generate such contradictory effects in ERα protein expression, our data is supported by evidence that EDCs display non-monotonic dose-response curves because hormones interact with and activate their receptors in a non-linear fashion, and this leads to a U-shaped or inverted U-shaped curve (Vandenberg et al., 2012; Vandenberg, 2014; Gore et al., 2015). EDCs could act in genomic and non-genomic nuclear receptor pathways, non-steroidal receptors, and ion channels, and its effects could be associated with tissue-specific epigenetic control and transcriptional coactivators (Gore et al., 2015). Additionally, a strong positive correlation between ERα protein expression levels and LTM was observed in the PFC of TBT mice (Supplementary Figure 2C). E2 is an ovarian steroid hormone that acts through the activation of ERs, playing a key role in normal brain function, neuroprotection, memory, and cognitive processes (Garcia-Segura et al., 2001; Marin et al., 2003; Arevalo et al., 2015; Tang et al., 2018). Fernandez et al. (2008) demonstrated that E2 (0.2 mg/kg) administration increases protein expression of phosphorylated ERK in the dorsal hippocampus from ovariectomized rats, increasing memory retention after an object recognition task in a training session. It has been suggested that E2 regulation of object recognition memory is mediated *via* activation of ERα in female mice (Frye et al., 2007; Phan et al., 2011; Pereira et al., 2014). ERα is the predominant ER in the mouse hippocampus and PFC, playing an important role in their normal function (Bailey et al., 2011). In addition, infusion of an ERα agonist in the dorsal hippocampus was shown to enhance novel object recognition memory in female mice *via* ERK-dependent mechanism (Boulware et al., 2013). Thus, our data suggest that disruptions of ERα protein levels in the PFC of female mice might be associated to impairments in recognition memory that we observed after TBT exposure, although a more specific approach is needed to confirm the relationship between estrogen receptor activation and cognitive alterations and to evaluate if other receptor types are involved in this mechanism.

In the current study, we found abnormal TBARS levels in the PFC of TBT-treated female mice, which is indicative of oxidative stress damage. Additionally, a strong negative correlation between TBARS levels and LTM was observed in the PFC of TBT mice (*p* = 0.0009; Supplementary Figure 2G). Previous studies have shown that TBT induces oxidative stress and subsequent dysfunction in several tissues, such as heart, artery, liver, kidney, testis, ovary, and brain (Ishihara et al., 2012, 2014; Mitra et al., 2013; Pereira et al., 2014; Coutinho et al., 2016; Kanimozhi et al., 2016; Ximenes et al., 2017; de Araújo et al., 2018). Mitra et al. (2015) reported that TBT increases reactive oxygen species (ROS) generation, leading to high lipid peroxidation and protein carbonylation in the cerebral cortex and hippocampus of male rats after a single TBT treatment. Moreover, TBT induced oxidative stress with astrocyte activation in rat cerebral cortex cells, resulting in extensive cortical damage and neuroinflammation (Mizuhashi et al., 2000). It has been shown that recognition memory relies on PFC activity, with disturbances in cortical function alone being sufficient to cause impairments, independent from hippocampal function (González et al., 2014; Tuscher et al., 2018). Also, disruption in hippocampal-prefrontal interaction leads to cognitive complications (Preston and Eichenbaum, 2013; Warburton and Brown, 2015; Eichenbaum, 2017). Thus, these findings are in accordance with our data, which suggest that TBT is capable of inducing abnormal PFC oxidative stress in female mice and subsequently impairing recognition memory, even though we did not find significant alterations in oxidative markers in the hippocampus. Although we found an increase in TBARS levels in the hippocampus, the results were not statistically significant, an issue we hypothesize that can be solved with a larger sample, since there are previously mentioned examples in literature of TBT-induced hippocampal lipid peroxidation. Nevertheless, we do consider the analysis of other oxidative parameters fundamental in order to further evaluate the extent of TBT-induced ROS damage.

In addition, abnormal reproductive cyclicity with excessive days in the MD phase is expected to lower circulating levels of estrogen and could explain the disturbances in ERα protein levels, and the increase in oxidative stress (de Araújo et al., 2018). Furthermore, a strong negative correlation between ERα protein expression and TBARS levels was observed in the PFC of TBT mice (*p* = 0.017; Supplementary Figure 2I). Therefore, TBT exposure could be at least in part responsible for the abnormal ERα protein expression and oxidative stress development, with those two phenomena being potential elements in the memory recognition impairment detected in this model.

## Conclusion

In conclusion, our data suggest that TBT exposure leads to recognition memory impairments, induces irregular STM, LTM, and total exploration time performances, reduces ERα protein expression in the PFC and hippocampus, and increases oxidative stress in the PFC. This study advances our understanding of TBT effects on recognition memory and elucidates some potential mechanisms underlying this process in female mice. Further studies, applying a variety of behavioral and biochemical techniques, would be useful to confirm a causal link between disruptions in ERα protein expression, oxidative stress, and recognition memory impairments in female mice, to determine the full extent of the neurotoxicity of TBT, and to develop intervention strategies aimed at preventing and suppressing the deleterious effects associated with TBT exposure.

## Data Availability Statement

The raw data supporting the conclusions of this article will be made available by the authors, without undue reservation.

## Ethics Statement

The animal study was reviewed and approved by Ethics Committee of Animal Use for Research (CEUA) Federal University of Espirito Santo.

## Author Contributions

IF, EM, JG and LR: conceptualization and writing (review and editing). IF, EM, and CC: methodology. IF, EM, CC, JG, and LR: data curation. IF: writing (original draft preparation). JG and LR: supervision, project administration, and funding acquisition. All authors contributed to the article and approved the submitted version.

## Conflict of Interest

The authors declare that the research was conducted in the absence of any commercial or financial relationships that could be construed as a potential conflict of interest.

## Supplementary Material

Supplementary Figure 1 Exposure to TBT results in abnormal estrous cycles in female mice. **(A)** A graphic representation of the estrous cycle in CON, TBT250, TBT500, and TBT750 mice as determined by vaginal cytology for 14 days (*n* = 4–6). **(B)** A graphic representation of the number of days in each stage of the estrous cycle and the total cycle length. P, proestrus; E, estrus; M-D, metaestrus-diestrus. ^*^*p* < 0.05 vs. CON (one-way ANOVA, followed by Bonferroni's test).

Supplementary Figure 2 Correlation between memory recognition performance, ERα protein expression, and oxidative stress in female mice. The values of ERα protein expression in the PFC **(A)** and hippocampus (HCP) **(B)** were correlated with STM performance. The values of ERα protein expression in the PFC **(C)** and HCP **(D)** were correlated with LTM performance. The values of TBARS levels in the PFC **(E)** and HCP **(F)** were plotted together with STM performance. The values of TBARS levels in the PFC **(G)** and HCP **(H)** were plotted together with LTM performance. The ERα protein expression in the PFC **(I)** was analyzed and correlated with TBARS levels. The ERα protein expression in the HCP **(J)** was analyzed and correlated with TBARS levels. Statistical significance (*p* ≤ 0.05) was tested using the Spearman's or Pearson's test for a non-Gaussian or Gaussian data distribution, respectively.
