# Supplementary figures and images for: Tributyltin Exposure Is Associated With Recognition Memory Impairments, Alterations in Estrogen Receptor α Protein Levels, and Oxidative Stress in the Brain of Female Mice
Source: Front Toxicol. 2021 Apr 9;3:654077. doi: 10.3389/ftox.2021.654077 (PMC8915859; doi:10.3389/ftox.2021.654077)

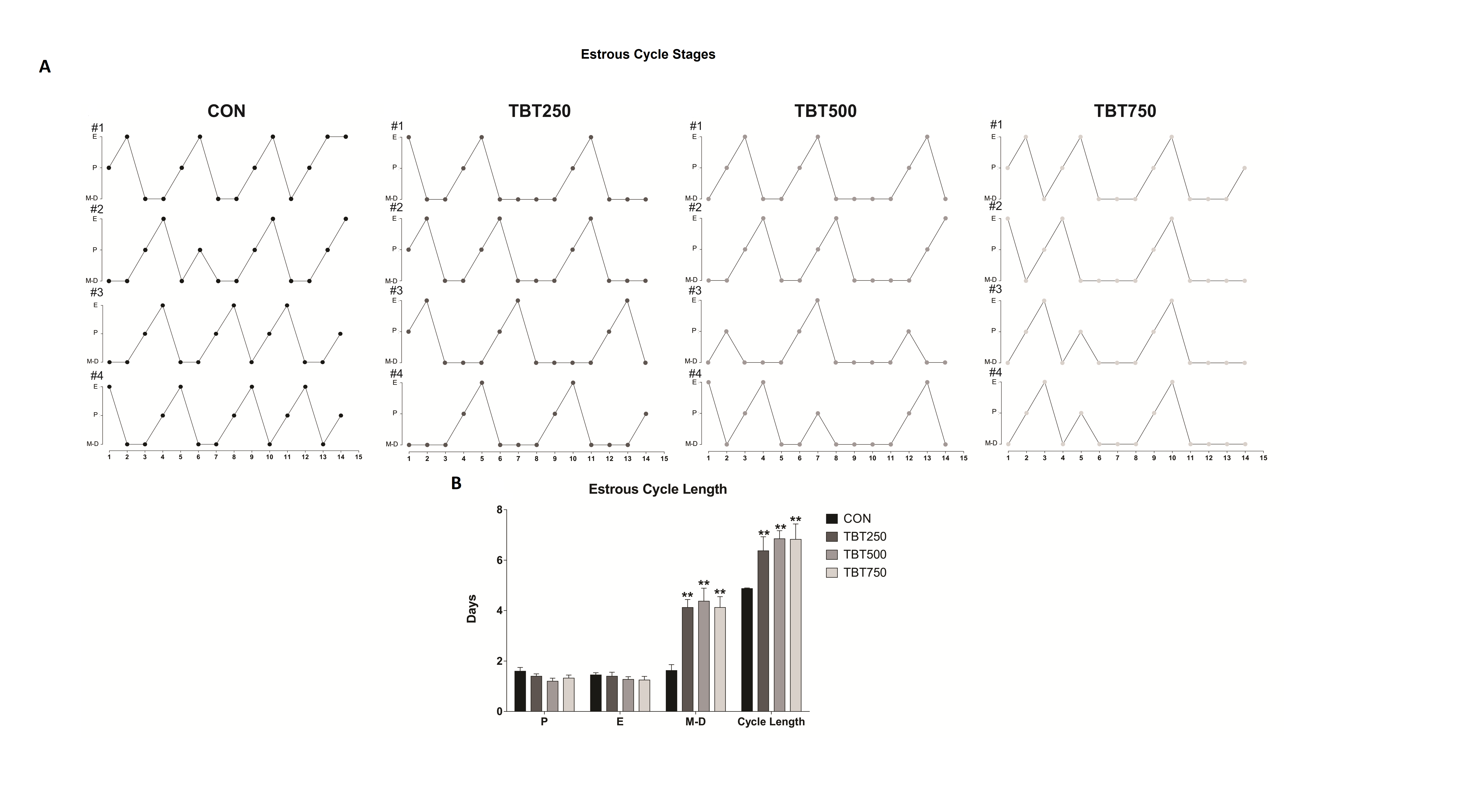

Supplement: Supplementary Figure 1 — Exposure to TBT results in abnormal estrous cycles in female mice. (A) A graphic representation of the estrous cycle in CON, TBT250, TBT500, and TBT750 mice as determined by vaginal cytology for 14 days (n = 4–6). (B) A graphic representation of the number of days in each stage of the estrous cycle and the total cycle length. P, proestrus; E, estrus; M-D, metaestrus-diestrus. *p < 0.05 vs. CON (one-way ANOVA, followed by Bonferroni's test). [file Image_1.TIF]

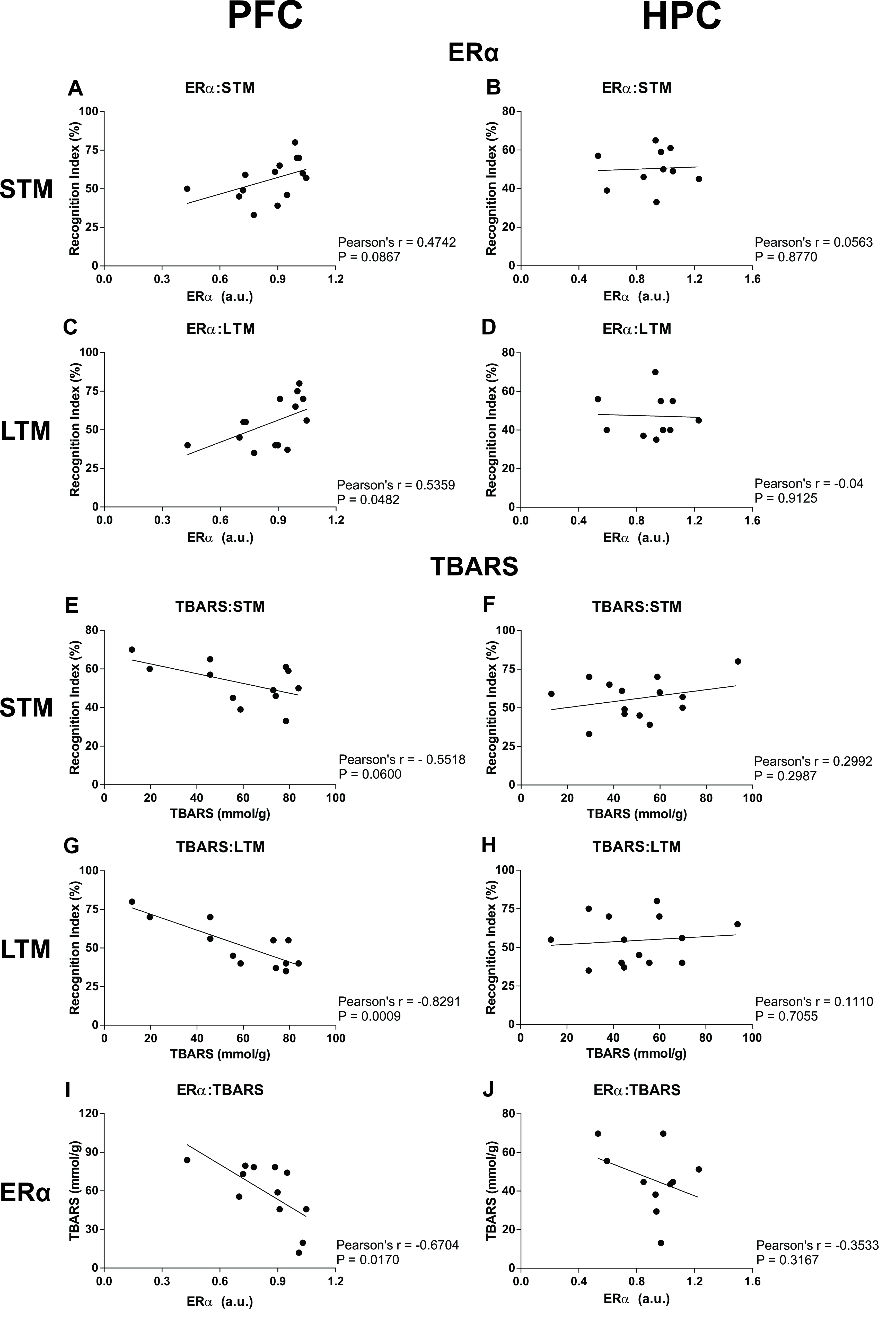

Supplement: Supplementary Figure 2 — Correlation between memory recognition performance, ERα protein expression, and oxidative stress in female mice. The values of ERα protein expression in the PFC (A) and hippocampus (HCP) (B) were correlated with STM performance. The values of ERα protein expression in the PFC (C) and HCP (D) were correlated with LTM performance. The values of TBARS levels in the PFC (E) and HCP (F) were plotted together with STM performance. The values of TBARS levels in the PFC (G) and HCP (H) were plotted together with LTM performance. The ERα protein expression in the PFC (I) was analyzed and correlated with TBARS levels. The ERα protein expression in the HCP (J) was analyzed and correlated with TBARS levels. Statistical significance (p ≤ 0.05) was tested using the Spearman's or Pearson's test for a non-Gaussian or Gaussian data distribution, respectively. [file Image_2.TIF]
